# Pediatric Abdominal X-rays in the Acute Care Setting – Are We Overdiagnosing Constipation?

**DOI:** 10.7759/cureus.7283

**Published:** 2020-03-15

**Authors:** Malik Muhammad Anwar ul Haq, Hernando Lyons, Madiha Halim

**Affiliations:** 1 Pediatrics, Essentia Health, Fargo, USA; 2 Pediatric Gastroenterology, Ascension St. John Hospital, Detroit, USA; 3 Internal Medicine, Sanford Health, Fargo, USA

**Keywords:** constipation, abdominal x-ray

## Abstract

Introduction

Constipation represents 3% of all office visits to pediatricians and 10% - 45% of consultations with pediatric gastroenterologists. It has been reliably established that the role of abdominal x-rays (AXR) in the diagnosis of constipation in pediatrics is limited; yet, significant overdiagnosis of constipation exists when plain abdominal x-rays are used in the acute setting for abdominal pain or to screen for other disorders. This results in loss of time, resources, exposure to unnecessary radiation, and potentially missing the primary diagnosis. The purpose of this study is to determine the sensitivity and specificity of AXR in diagnosing constipation in the acute setting.

Objectives

To determine 1) the sensitivity and specificity of plain AXR in the diagnosis of constipation and 2) the effect of age, race, gender, comorbid conditions, and practice setting on the diagnosis of constipation.

Methods

This study was a historical cohort study of children (two to 18 years of age) who were seen at Ascension St. John Children’s Hospital between March 2015 - March 2018 and who had a plain AXR performed during an emergency department (ED) visit or inpatient stay. If AXR results contained keywords, such as “constipation,” “stool load,” “fecal retention,” and “fecal load,” the ambulatory medical record, Athena® (http://www.athenahealth.com), was searched to determine if the child had an ambulatory visit in the ensuing 45 days. Chart review was conducted to assess if the diagnosis of constipation was later confirmed by history and physical examination by a pediatrician or gastroenterologist at that visit. By comparing data from both encounters, the sensitivity and specificity of plain AXR in diagnosing constipation was assessed. All data were analyzed using the Statistical Package for Social Sciences (SPSS), v. 25.0 (IBM SPSS Statistics, Armonk, NY) and a p-value of 0.05 or less was considered to indicate statistical significance.

Results

Over the three-year study period, 1,383 AXRs were performed on 1,116 patients. The sensitivity of AXR in the diagnosis of constipation was 73.8%, specificity 26.8%, positive predictive value 46.4%, and negative predictive value of 54.3%. Pediatric gastroenterologists were more likely to diagnose constipation (63.2%) compared to pediatricians (41.4%) and pediatric surgeons (33.3%) (p = 0.04).

Conclusions

AXRs are not a reliable means of diagnosing constipation. Overall, we found similar sensitivity and specificity of AXR in diagnosing constipation compared to previous studies. Yet, our study gives new insight into the practices around diagnosing constipation in a single-center community hospital pediatric acute setting and the radiology department. This further emphasizes the need to review current practices and impart more education both in the acute care setting and radiology department.

## Introduction

Constipation represents 3% of all office visits to pediatricians and 10% to 45% of consultations with pediatric gastroenterologists. In primary care settings, 50% of children with abdominal pain are diagnosed with constipation. Although abdominal radiographs (AXRs) lack validity and reliability when employed in the context of pediatric abdominal pain and have a limited ability to predict constipation, they are performed in 75% of children diagnosed with constipation in a pediatric emergency department (ED). 

Multiple scoring systems have been devised to assess fecal retention on abdominal x-rays objectively. These include the Barr, Leech, and Blethyn scoring systems. Although they may provide a more reliable and objective method of evaluating abdominal x-rays for constipation, there is low utilization of these scores among radiologists. Subjective experience-based evaluation is still the predominant method of evaluating abdominal x-rays in pediatrics. This results in significant interobserver variability, unreliability, and lack of reproducibility. Also, there is insufficient evidence to support the validity of the scoring systems to be used widely.

It has been reliably established that the role of abdominal x-rays in the diagnosis of constipation in pediatrics is limited. However, significant overdiagnosis of constipation exists when plain abdominal x-rays are used in the acute setting for abdominal pain or to screen for other disorders. This results in loss of time, resources, exposure to unnecessary radiation, and potentially missing the primary diagnosis.

The purpose of this study was to determine the sensitivity and specificity of abdominal x-rays in the diagnosis of constipation in children. 

## Materials and methods

This study was a historical cohort study of children, ages two to 18 years, who had a plain abdominal x-ray performed for the first time at Ascension St. John Children's Hospital between March 2015 and March 2018 during an ED visit or inpatient stay. The electronic medical record, eCare®, was reviewed for demographic, clinical, and radiologic data. When radiology results contained keywords, such as “constipation,” “stool load,” “fecal retention,” and “fecal load,” the child was classified as being diagnosed with constipation at the time of the x-ray. For all patients who had an x-ray, we queried the ambulatory medical record, Athena® (http://www.athenahealth.com), to determine if the child had an ambulatory visit in the ensuing 45 days. Chart review was conducted to assess whether the diagnosis of constipation was later confirmed by history and physical examination by a pediatrician or gastroenterologist at that visit. By comparing data from both encounters, the sensitivity and specificity of plain AXRs in diagnosing constipation was assessed.

We included children between two to 18 years of age who had a plain abdominal radiograph done during an inpatient stay or emergency department (ED) visit between March 2015 - March 2018 and had a follow-up visit with a pediatrician, pediatric gastroenterologist, or pediatric surgeon at our health system for clinical assessment within 45 days. In regard to children under the age of two years, those patients who had prior abdominal radiographs at our institution and patients without ambulatory follow-up were excluded from the study.

Data were collected on age, sex, race, and indication for the abdominal x-ray (radiologist-reported diagnosis of constipation/stool/bowel retention, physician diagnosis of constipation on post-discharge follow-up using history and physical examination alone, comorbidities, and alternative diagnosis or additional diagnosis).

Descriptive statistics were generated to characterize the study group. Continuous variables were described using the mean with standard deviation or median with interquartile range (IQR) for continuous variables. Categorical variables were described as frequency distributions. The sensitivity and specificity of plain x-rays versus the final diagnosis from the pediatrician, pediatric gastroenterologist, or pediatric surgeon were calculated. Demographic and clinical factors associated with the diagnosis of constipation were assessed using the Student’s t-test and the Chi-square test. All data were analyzed using the Statistical Package for Social Sciences (SPSS), v. 25.0 (IBM SPSS Statistics, Armonk, NY) and a p-value of 0.05 or less was considered to indicate statistical significance.

This study was approved by the Ascension St. John Hospital Institutional Review Board (approval #1251825).

## Results

A total of 1,116 charts were reviewed over the three-year study period of which 132 were included in the study (Table 1). The mean age of the study group was 10.3 ± 4.3 years, with a range of two to 18 years. For the 1,116 patients, 1,383 AXRs were performed (Table 2). The sensitivity of AXR in the diagnosis of constipation was calculated to be 73.8%, specificity of 26.8%, a positive predictive value of 46.4%, and a negative predictive value of 54.3% (Tables 3-4). 

**Table 1:** X-rays Meeting Inclusion Criteria (Keywords) Out of Total X-rays Included

Patients meeting inclusion criteria	Number (n)(percentage)
Total X-rays included in the study	132
X-rays containing keywords	97 (74%)
X-rays without keywords	35 (26%)

**Table 2:** Data Characteristics

Data Characteristics	Number (n)
Total X-rays	1,383
Total patients	1,116

**Table 3:** Percentage of Patients Diagnosed with Constipation by Outpatient Physicians p = 0.94

Abdominal X-Ray Result	Diagnosed as Constipation at Outpatient Visit (%)
X-rays containing keywords	46.4%
X-rays without keyword	45.7%

**Table 4:** Test Characteristics

Test characteristics	Percentage
Sensitivity	73.8%
Specificity	26.8%
Positive predictive value	46.4%
Negative predictive value	54.3%

The indication for AXR was abdominal pain in 68.2% of patients, nausea/vomiting in 22.7% of patients, constipation in 26.5%, and foreign body in 1.5% of patients. When the indication was nausea/vomiting or constipation, patients were more likely to be diagnosed with constipation clinically (p = 0.004 and 0.007, respectively). Younger patients were more likely to be reported as constipated on AXR (p = 0.007) and diagnosed by a physician as constipation (p = 0.01). Pediatric gastroenterologists (63.2%) were more likely to diagnose constipation compared to pediatricians (61.4%) and pediatric surgeons (33.3%) (p = 0.04). On the other hand, race, gender, the presence of food allergies, comorbid conditions (such as anorectal anomalies), neuromuscular disorders, and history of prematurity did not significantly affect the diagnosis of constipation by AXR. 

## Discussion

In this historical cohort study, it was found that approximately one in every two children labeled as constipated on AXR were not constipated, whereas one in four patients who presented to our ED with complaints of constipation had an AXR done just to confirm constipation [1-6]. Hence, the excess of AXR exposes patients to unnecessary radiation and puts the health-related economic burden on the country [7-9].

Reuchlin-Vroklage et al. reported similar results in their systemic review. Their study concluded against the use of imaging to confirm the doubtful diagnosis of constipation [10]. In another systemic review, Berger et al. reported insufficient data to support the diagnosis of constipation radiologically [11]. On the other hand, Leech et al. recommended the use of a scoring system in AXR to diagnose constipation [9]. They also recommended that radiography should only be used in the case of intractable constipation to reduce exposure to ionizing radiation.

In our study, the ED had more visits (65%) compared to the inpatient setting (35%), but there was no significant difference found in the diagnosis of constipation on AXR. Moreover, patients with nausea/ vomiting or constipation as an AXR indication were more likely to be diagnosed with constipation clinically. These findings were contrary to findings reported by Beckmann et al. in their study where they concluded against any clinical variable in the identification of abdominal pain or constipation that has been proven radiographically [5]. 

The strengths of our study were the historical cohort design and that we were able to review all the radiology records for children who fit our study criteria. The study was conducted at a large urban teaching hospital that serves a diverse group of patients. The primary limitation is that we did not know what happened to patients who did not have a follow-up ambulatory visit at our site. We also could not objectively determine the reliability in interobserver differences in radiology reporting based on individual preference and experience. There was also some lack of standardization in outpatient physician reporting of the history and physical examination findings [12-14].

## Conclusions

We found similar sensitivity and specificity of AXR in diagnosing constipation compared to previous studies. AXRs are not a reliable means of diagnosing constipation and inadvertently add to health care costs, radiation exposure, missed alternate diagnoses, and unnecessary treatment. While the lack of the reliability of AXR in diagnosing constipation has been well-established, our study did give new insight into the practices around diagnosing constipation in a single-center community hospital pediatric acute setting and radiology department. We found a significant number of overdiagnoses of constipation using just an AXR which was later not clinically proved.
